# Comparative Microbiomics of Tephritid Frugivorous Pests (Diptera: Tephritidae) From the Field: A Tale of High Variability Across and Within Species

**DOI:** 10.3389/fmicb.2020.01890

**Published:** 2020-08-11

**Authors:** Maarten De Cock, Massimiliano Virgilio, Peter Vandamme, Kostas Bourtzis, Marc De Meyer, Anne Willems

**Affiliations:** ^1^Royal Museum for Central Africa, Tervuren, Belgium; ^2^Laboratory of Microbiology, Department of Biochemistry and Microbiology, Faculty of Sciences, Ghent University, Ghent, Belgium; ^3^Insect Pest Control Laboratory, Joint Food and Agriculture Organization of the UnitedNations/International Atomic Energy Agency (FAO/IAEA) Division of Nuclear Techniques in Food and Agriculture, Vienna, Austria

**Keywords:** gut microbiomics, insects, *Bactrocera*, *Zeugodacus*, *Ceratitis*

## Abstract

The family Tephritidae includes some of the most notorious insect pests of agricultural and horticultural crops in tropical and sub-tropical regions. Despite the interest in the study of their gut microbiome, our present knowledge is largely based on the analysis of laboratory strains. In this study, we present a first comparative analysis of the gut microbiome profiles of field populations of ten African and Mediterranean tephritid pests. For each species, third instar larvae were sampled from different locations and host fruits and compared using 16S rRNA amplicon sequencing and a multi-factorial sampling design. We observed considerable variation in gut microbiome diversity and composition both between and within fruit fly species. A “core” microbiome, shared across all targeted species, could only be identified at most at family level (Enterobacteriaceae). At genus level only a few bacterial genera (*Klebsiella*, *Enterobacter*, and *Bacillus*) were present in most, but not all, samples, with high variability in their relative abundance. Higher relative abundances were found for seven bacterial genera in five of the fruit fly species considered. These were *Erwinia* in *Bactrocera oleae*, *Lactococcus* in *B. zonata*, *Providencia* in *Ceratitis flexuosa*, *Klebsiella*, and *Rahnella* in *C. podocarpi* and *Acetobacter* and *Serratia* in *C. rosa*. With the possible exception of *C. capitata* and *B. dorsalis* (the two most polyphagous species considered) we could not detect obvious relationships between fruit fly dietary breadth and microbiome diversity or abundance patterns. Similarly, our results did not suggest straightforward differences between the microbiome profiles of species belonging to C*eratitis* and the closely related *Bactrocera/Zeugodacus*. These results provide a first comparative analysis of the gut microbiomes of field populations of multiple economically relevant tephritids and provide base line information for future studies that will further investigate the possible functional role of the observed associations.

## Introduction

Plants are able to produce a wide variety of allelochemicals that act as deterrents against phytophagy. The capability of phytophagous insects to overcome these toxic compounds is strictly associated with the insect feeding preferences and host plant range. This is thought to represent an important evolutionary process promoting insect speciation and, ultimately insect-plant co-evolution (Aluja and Norrbom, 1999; Després et al., 2007; Winkler and Mitter, 2016; Chen et al., 2017). The family of the Tephritidae (Diptera), commonly referred to as fruit flies, consists worldwide of more than 4500 species distributed over 500 genera (White and Elson-Harris, 1992; Uchôa, 2012). Multiple species are found on all continents, excluding Antarctica, but they mainly thrive in tropical and sub-tropical environments. Although the majority infests the seed-bearing organs of plants, about half of the 4500 fruit fly species use the actual fruits for their own reproduction. Eggs are laid in ripening fruits and the different stages of larval development take place within the fruit. Larvae leave the fruit before pupation, pupate in the soil in order to emerge and become adult fruit flies (Christenson and Foote, 1960; Aluja and Norrbom, 1999). This larval development causes damage to the fruit, both directly by damaging the fruit tissue, and indirectly by accelerating the rotting process and increasing infestation by other insects, fungi and bacteria (Pierre, 2007; Badii et al., 2015; Qin et al., 2015; Alvarez et al., 2016). Fruit flies are found in both wild and commercial fruits and because of this, infestations by fruit flies can have huge economic impacts on the agricultural sector.

As many other phytophagous insects, tephritids can differ widely in their degree of host plant specialization and attack only one host plant species (monophagous flies), only one genus of host plant species (stenophagous), different genera within the same family (oligophagous) or a wide range of hosts belonging to several unrelated plant families (polyphagous). However, the functional classification based on feeding preferences is sometimes ambiguous as flies are also sporadically recorded not only on their “natural” host plants (*sensu* Aluja and Mangan, 2008) but also, and sporadically, on “unconventional” hosts (De Meyer et al., 2015; Hafsi et al., 2016). Previous phylogenetic research suggested that the evolutionary relationships observed in fruit flies might be related to their feeding preferences and host plant specialization (Virgilio et al., 2009). In particular, strong specialization on host plant species (i.e., monophagy and stenophagy) seems to be associated with the capacity to metabolize toxic secondary compounds of the host plant enabling fruit flies to exploit hosts inaccessible to polyphagous flies (Erbout et al., 2011; Pavlidi et al., 2013, 2017; Ben-Yosef et al., 2015). Because of the overall importance of microbial symbionts, it has been hypothesized that microbes might play a crucial role in shaping the dietary range and host plant specialization of herbivorous insects (microbial facilitation hypothesis (Janson et al., 2008; Brucker and Bordenstein, 2012; Douglas, 2013; Hansen and Moran, 2014; Hammer and Bowers, 2015). However, it is not entirely clear how important the relative contribution of microbial symbionts is in facilitating host plant shifts and host plant specialization compared to other processes, including the capacity of insects to produce plastic metabolic responses when changing host plant (Pfennig et al., 2010).

In recent years, an increasing number of studies have focused on the gut microbiome of tephritid fruit flies (Lauzon et al., 2000; Bourtzis and Miller, 2003; van den Bosch and Welte, 2016; Cheng et al., 2017; Ras et al., 2017; Cáceres et al., 2019). Largely thanks to the emergence of high throughput sequencing (HTS) techniques which facilitated the analysis of complex assemblages generally including thousands of Amplicon Sequence Variants (ASVs) (Wang A. et al., 2014; Wang H. et al., 2014; Andongma et al., 2015). As observed in other insects, evidence has emerged that bacteria help to overcome pesticides (Cheng et al., 2017) and boost host defenses (Ben-Yosef et al., 2015) or generally increase longevity of fruit flies (Niyazi et al., 2004; Behar et al., 2008a; Hamden et al., 2013; Sacchetti et al., 2013). Complex relationships may exist between the feeding strategy and the gut microbiome with the general expectation that monophagous flies might harbor a more specialized gut microbiome, while polyphagous species should harbor a more diverse and less specialized gut microbiome. Precedence for this kind of relationship was found in *Bactrocera oleae*, a strict monophagous species. Studies have unveiled a close evolutionary relationship between *B. oleae* and the bacterial species *”Candidatus* Erwinia dacicola*”* (Capuzzo et al., 2005; Estes et al., 2009). It has been shown that this bacterial species has an important role in facilitating the digestion of olives, and that its absence may strongly reduce survival rate of *B. oleae* in the field (Ben-Yosef et al., 2008, 2015).

Most of the currently available research on tephritid gut microbiomics focuses on fruit fly laboratory populations (i.e., fed with artificial diets) and often aims at investigating the optimal rearing conditions for species of interest for the sterile insect technique (SIT) (Augustinos et al., 2015, 2019; Kyritsis et al., 2017, 2019; Asimakis et al., 2019) while the composition and levels of variability of microbiome profiles of wild tephritid flies are far less known.

A number of studies have targeted one (Wang H. et al., 2014; Deutscher et al., 2018; Malacrinò et al., 2018; De Cock et al., 2019) or a few (Morrow et al., 2015) fruit fly species and compared the microbiomes of wild and laboratory populations. Other studies investigated relationships between the microbiome composition of a single fruit fly species and the host plant attacked (Zaada et al., 2019) or the geographic origin of larvae (Hadapad et al., 2015; Koskinioti et al., 2019). Regardless of that, there is still the need for a better understanding of patterns of variability of microbiome profiles in wild flies, and studies providing wide inter- and intra-specific comparisons in field conditions are, to our knowledge, currently missing.

The present study aimed at providing a first wide-range comparative analysis of the microbiome profiles of tephritid flies as observed under field conditions (i.e., from larvae sampled while feeding on their natural host plants). In this respect, we characterized the microbiome profiles of representative monophagous, stenophagous, oligophagous and polyphagous species from three economically important genera in the Mediterranean region and Sub-Saharan Africa.

Due to the relatively high heterogeneity previously observed for the microbiome of both laboratory and field populations of *Ceratitis capitata* (De Cock et al., 2019) we decided to characterize the intra-specific variability of microbiome assemblages by considering field populations from replicated sampling sites and host plants. This approach aimed at verifying the presence of particular groups of gut symbionts consistently associated to the targeted fruit fly species while disentangling the effects of geographic variability and host plant choice.

## Materials and Methods

### Sample Collection and Experimental Setup

We targeted three tephritid genera of economic relevance (*Bactrocera, Zeugodacus*, and *Ceratitis*) including ten representative fruit fly species [*B. dorsalis* (Hendel), *B. oleae* (Rossi), *B. zonata* (Saunders), *Z. cucurbitae* (Coquillett), *C. capitata* (Wiedemann), *C. cosyra* (Walker), *C. flexuosa* (Walker), *C. podocarpi* (Bezzi), *C. quilicii*, De Meyer, Mwatawala & Virgilio, and *C. rosa*, Karsch]. The species selection covered a range of feeding strategies including monophagy (*B. oleae, C. flexuosa*), stenophagy (*C. podocarpi*), oligophagy (*Z. cucurbitae*), and polyphagy (at increasing levels of polyphagy: *C. cosyra, B. zonata, C. quilicii, C. rosa, C. capitata*, and *B. dorsalis*).

A first part of this study was based on a balanced sampling design (Supplementary Table S1), as required for ANOVA/PERMANOVA (see below). Here we considered five fruit fly species (*B. dorsalis*, *Z. cucurbitae, B. oleae*, *C. capitata*, and *C. quilicii*) and, for each of them, the geographic variability of microbiome assemblages was estimated by collecting samples from two arbitrarily chosen locations in two different African or European countries. Similarly, intraspecific variability associated to host-plant choice was estimated by collecting three replicate samples in fruits from two randomly chosen host plant species at each location (see Supplementary Tables S1, S2). As in De Cock et al. (2019), we tried to reduce inter-individual variability by pooling, for each sample, the dissected guts of five third instar larvae. This first balanced experiment (dataset A) included a total of 60 samples as obtained from 300 dissected guts.

This dataset was then expanded with 33 additional samples from the ten fruit fly species listed above, collected from additional host plants and sampling locations (see details in Supplementary Tables S1, S2). This allowed considering a larger dataset (dataset B) including a total of 93 samples obtained from the dissection of 465 larval guts which was used for a wider range of statistics (see below).

### Laboratory Procedures

After collection in the field or in the rearing facilities of partner Institutions (see Acknowledgments), larvae were immediately stored in 70% ethanol before being transferred to the Royal Museum for Central Africa (Tervuren, Belgium). There, individual larvae were rinsed again in 70% ethanol for 30 s and washed in sterile phosphate buffed saline (PBS) water. The complete gut was dissected under sterile conditions as detailed in De Cock et al. (2019). Although we acknowledge that the use of diluted rather than absolute ethanol as a killing and preserving agent is suboptimal and might have affected the gut microbial community, contributing to the variability of microbiome profiles across experimental replicates (see De Cock et al., 2019). We eventually considered this was the only suitable methodological approach to keep the larval tissues soft and allow dissections. From each gut, DNA was extracted using the Qiagen DNAeasy kit, as per manufacturer’s instructions. After DNA extraction the identity of each larva was confirmed via DNA barcoding as described in Virgilio et al. (2012) and DNA concentrations were quantified using a Qubit fluorometer (Thermo Fisher Scientific). We only selected DNA extracts from larvae having a correct identification and DNA concentrations higher than 1 ng/μl. For each sample, three replicates were prepared, each consisting of the pooled DNA extracts from five individual larvae (normalized DNA concentrations). This way about 951 larvae were processed from which 358 DNA extracts needed to be rejected (199 wrong identification, 82 failed identification and 77 DNA concentration to low). From the remaining 593 DNA extract, 465 extracts were selected to create our pooled samples. A mock community was composed consisting of the DNAs of 18 pure bacterial strains (see Supplementary Table S3) obtained from the BCCM/LMG Bacteria Collection^1^. The species were selected based on literature reports of their occurrence in fruit fly guts. Bacterial strains were individually grown following the BCCM/LMG catalog instructions. DNA was extracted using the Qiagen DNAeasy kit, and mixed in equal concentrations (DNA concentration: 10 ng/μl). This mock sample and a blank sample were also included in sample preparation and sequencing protocol as, positive and negative control.

Genomic library preparation for 16S rDNA metagenomics relied on the Nextera XT kit (Illumina, 2016). In a first step, the primers 341F and 806R (insert size 465 bp), targeting the V3–V4 region of the 16S ribosomal RNA (Takahashi et al., 2014), were used to amplify the targeted region of the bacterial 16S rRNA, simultaneously two Illumina sequencing adapters were attached to the target DNA fragment. In a second step, dual-index barcodes were attached to the Illumina sequencing adapters. If needed, this second step was repeated to increase DNA yield. A final check of quality and fragment size was performed via an Agilent 2100 Bioanalyzer. Libraries were sequenced on an Illumina MiqSeq platform (300 bp paired end sequencing) by Macrogen (Amsterdam).

### Data Analysis

Read quality was evaluated using FastQC (Andrews, 2014). The pipeline DADA2 (Callahan et al., 2016), implemented in R, was used for data filtering. This pipeline is based in a self-learning algorithm, which sets up a parametric error model that fits the raw data. This model is then used to infer sequencing error. In DADA2, raw reads were trimmed, demultiplexed, filtered and paired (Callahan et al., 2016). Processed reads were assigned to Amplicon Sequence Variants (ASVs) according to the Bayesian classifier method implemented by DADA2 (Wang et al., 2007) (percentage of identity = 97% similarity, *p*-min-consensus = 0.51). Taxonomic assignment of ASV relied on the Silva v132 (26) database. The robustness of the assignment was double-checked against the RDP (Cole et al., 2014) and Greengenes databases (DeSantis et al., 2006, data not shown). The full analytical pipeline is detailed in Supplementary Table S4. As in De Cock et al. (2019), before analyses, single- and doubletons reads were filtered out to reduce possible biases due to sequencing error. For comparative analysis, normalized data, based on the median sample number of reads, was used (de Cárcer et al., 2011).

The data were processed in both univariate and multivariate frameworks. The effects of Fruit Fly Species (FFSp), Location (Lo), and Host plant (Ho) on univariate patterns of alpha diversity, as estimated by the Simpson index D (Sagar and Sharma, 2012), were tested via Analysis of Variance (ANOVA) (Underwood, 1997). Comparisons of multivariate patterns were done by using Permutational Multivariate Analysis of Variance (PERMANOVA, Anderson, 2017) and Permutational Multivariate Analysis of Dispersion (PERMDISP, Anderson, 2001). We used PERMANOVA to test differences in the relative abundance of ASVs (2749 in total, see section “Results”), while, as the PERMDISP routine of Anderson (2001) can only be implemented on a maximum of 500 variables, this analysis was implemented on the relative abundance of genera (401 in total, see section “Results”). In order to reduce differences in scale among variables while preserving information about taxa proportions, we transformed the multivariate data following Clarke (1993). This approach allowed reducing the importance of dominant, compared to the less abundant, taxa and to better identify more subtle changes in the abundance of non-dominant species. We compared the possible impact of data transformation by implementing both (1) presence-absence transformation (as an example of extreme transformation severely affecting abundance proportions) and (2) fourth-root transformation (as an example of less aggressive transformation, of common use in community ecology. For both ANOVA and PERMANOVA a three-way factorial setup was adopted with fruit fly Species (FFSp) as a fixed, orthogonal factor and Location [Lo(FFSp)] and Host Plant [Ho(FFSpxLo)] as random, nested factors. For PERMDISP, that only allows two-way designs (Anderson, 2006), we tested the effects of FFSp, and either [Lo(FFSp)] or [Ho(FFSp)]. *A posteriori* pairwise comparisons of significant factors were implemented via Tukey’s Honestly Significant Difference (HSD) test (Abdi and Williams, 2010) for ANOVA and permutational t-statistics for PERMANOVA and PERMDISP (Anderson, 2001, 2017). Probability values of repeated *a posteriori* tests were corrected for Type I errors using the False Discovery Rate procedure (Benjamini and Hochberg, 1995) with experiment-wise probability *p* = 0.05. In order to increase the power of the multivariate *a posteriori* test (Underwood, 1997), we increased the number of permutable units (Anderson, 2017) by pooling together the replicates of non-significant terms. Following de Cárcer et al. (2011), we repeated multivariate tests on both data fourth-root transformed to the median and presence/absence data. The analysis of presence/absence data allowed stressing the possible effects of less abundant taxa.

Further investigation of the gut microbiome composition was done using the packages Phyloseq (McMurdie and Holmes, 2013), Vegan (Oksanen et al., 2019), and ggplot2 (Wickham, 2009), as implemented in R version 3.1.0. Principal Coordinates Analyses (PCoAs) based on Bray-Curtis distance (Bray and Curtis, 1957) were calculated for both fourth-root transformed data and presence/absence data. PCoAs for separated species were not incorporated due to the relatively small proportion of variance represented in PCoAs and to the relatively small sample size of samples available for each host and location. ASVs were pooled based on the bacterial genera and the percentage contribution of each of these genera to the average Bray-Curtis dissimilarity between fruit fly species was calculated using SIMPER (Clarke, 1993) on standardized, untransformed data. A permutational test based on 10,000 iterations was used to identify bacterial genera significantly differing between fruit fly species. Repeated permutational tests were corrected using FDR (Benjamini and Hochberg, 1995) at an experiment-wise *p* < 0.01. The results of SIMPER pairwise tests were summarized by considering only those bacterial genera (a) significantly differing between fruit fly species and (b) with an average contribution to dissimilarity higher than 5%.

## Results

The MiSeq Illumina run produced more than 19 × 10^6^ paired-end (PE) reads (average per sample = 213185.07; *SD* = 72270.58). Following quality assessment in FastQC (Andrews, 2014), the forward and reverse reads were trimmed at respectively 240 and 210 bp. Based on read quality, a strict error rate (max N’s = 0, max error rate = 1, see Supplementary Table S4) was applied in DADA2. After filtering, demultiplexing and merging about 5.4 × 10^6^ reads, 2749 unique ASVs were identified. The analysis of reads from the positive control did not suggest relevant biases while reads corresponding to 11 ASVs detected in the negative control (see Supplementary Table S5) were eliminated from the datasets to avoid possible biases.

The 2749 ASVs were assigned to 401 genera belonging to 142 different families and 22 phyla (Supplementary Figure S1). Of these phyla, Proteobacteria was by far the most dominant, representing 89.25% of all reads, followed by Firmicutes (8.43%), Bacteroidetes (0.95%), Actinobacteria (0.83%), Epsilonbacteraeota (0.22%), and Tenericutes (0.18%). The remaining phyla represented only about 0.01% of total reads. The phylum Proteobacteria consisted of 62 bacterial families, mainly represented by Enterobacteriaceae (65.60% of all reads), Acetobacteraceae (16.72%), Rhizobiaceae (3.37%), and Burkholderiaceae (0.69%) (Supplementary Figure S2). The phylum Firmicutes consisted of 27 bacterial families, mainly represented by Leuconostocaceae (4.16%), Streptococcaceae (2.60%), and Lactobacillaceae (0.52%) (Supplementary Figure S2). The phylum Bacteroidetes consisted of 23 bacterial families, mainly represented by Weeksellaceae (0.57%), Dysgonomonadaceae (0.14%), and Flavobacteriaceae (0.10%) (Supplementary Figure S2). The phylum Actinobacteria consisted of 33 bacterial families, mainly represented by Microbacteriaceae (0.29%) and Corynebacteriaceae (0.27%) (Supplementary Figure S2). The remaining phyla are all represented by only one or a few bacterial families. Of the above-mentioned phyla, only Proteobacteria was present in every sample. The phylum Firmicutes was present in almost all samples (> 90%) but had a very low abundance in some samples. The phyla Bacteroidetes and Actinobacteria were present in most samples, respectively, 64 and 73%. All remaining phyla were present in less than 25% of the samples. At bacterial family level, only the family of Enterobacteriaceae was present in all samples. Of the remaining bacterial families only Moraxellaceae, Burkholderiaceae, Streptococcaceae, Acetobacteraceae, Bacillaceae, Corynebacteriaceae, Leuconostocaceae and Staphylococcaceae were present in more than half of the samples. At bacterial genus level there were no genera present in every sample and only a few genera were present in the majority of the samples, including *Klebsiella* (96.43% of samples), *Bacillus* (96.43%), *Enterobacter* (92.86%), and *Acinetobacter* (89.29%). However, high variability between samples, and replicates could be observed with no bacterial genera dominant across all samples. A detailed overview of the most abundant bacterial genera for each fruit fly species can be found in Supplementary Table S11. PERMANOVA on fourth-root transformed data (Dataset A, Table 1, and Supplementary Table S6) showed that the gut microbiome composition significantly differs between fruit fly species (*p* < 0.01) and host plants (*p* < 0.001). PERMANOVA on presence/absence data (Table 1 and Supplementary Table S7) could also detect a significant effect of location, suggesting that the gut microbiome of conspecific samples from different locations differs with respect to the less abundant ASVs. The *post hoc* tests on fourth-root transformed data (pooled for location) showed significant differences in all pairwise comparisons with *B. oleae* as well as between *B. dorsalis* and all other species but *C. capitata*, between *Z. cucurbitae* and all other species but *C. capitata*, and between *C. quilicii* and all other species but *C. capitata* (Supplementary Table S4). The *post hoc* comparison also provided indications on variability of the gut microbiome composition of the same species when feeding on different host plants. While the microbiome profiles of *Z. cucurbitae* and *C. quilicii* did not show significant variation across host plants, in both *B. dorsalis* and *C. capitata*, we found differences in most (all but one) pairwise comparisons (Supplementary Table S6). *Post hoc* comparison on presence/absence data did not reveal any significant effect (Supplementary Table S7).

**TABLE 1:** PERMANOVA (fourth-root transformed and presence/absence data; dataset A) testing differences in the microbiome profiles (2,749 ASVs considered) of five fruit fly species (FFSp, *B. dorsalis, Z. cucurbitae, B. oleae, C. capitata, C. quilicii)* sampled in two locations [Lo(FFSp)] from two host plants within each location [Ho(FFSpxLo)].

	*df*	*MS*	*F*	*p*-value	
**Fourth-root transformed data**					
FSp	4	23502.690	3.521	0.001	**
Lo(FFSp)	5	6675.461	1.220	0.189	n.s.
Ho(FFSpxLo)	10	5472.781	2.548	0.000	***
Residual	40	2148.190			
Total	59				
**Presence/Absence data**					
FFSp	4	18961.396	2.557	0.000	***
Lo(FFSp)	5	7414.919	1.345	0.041	*
Ho(FFSpxLo)	10	5514.679	2.733	0.000	***
Residual	40	2018.200			
Total	59				

***p < 0.001, **p < 0.01, *p < 0.05, n.s.p > 0.05.

Pooling the taxonomically assigned ASVs for the balanced experiment (Dataset A), by genus resulted in a dataset of 401 distinct bacterial genera. On both fourth-root transformed and presence/absence data, PERMDISP revealed significant effects of fruit fly species (*p* < 0.01) and host (*p* < 0.001) on multivariate dispersion (Table 2 and Supplementary Tables S8, S9). Although the average dissimilarity between replicates in *B. oleae* (as calculated from fourth-root transformed data) was lower than in all other species, we did not observe significant differences in the *post hoc* comparisons between species (Table 2, Supplementary Tables S8, S9).

**TABLE 2:** PERMDISP (fourth-root transformed and presence/absence data; dataset A) testing differences in the microbiome profiles (401 bacterial genera considered) of five fruit fly species (*B. dorsalis*, *Z. cucurbitae*, *B. oleae*, *C. capitata*, *C. quilicii*) sampled from four different host plants.

	*df*	*MS*	*F*	*p*	
**Fourth-root transformed data**					
FFSp	4	1555.209	6.668	0.004	**
Ho(FFSp)	15	233.240	6.179	0.000	***
Residual	40	37.745			
Total	59				
**Presence/Absence-data**					
FFSp	4	749.530	7.637	0.003	**
Ho(FFSp)	15	98.143	1.822	0.045	**
Residual	40	53.860			
Total	59				

**Average within-group dissimilarities**	**Fourth-root transformed data**		**Presence/Absence-data**		

*B. dorsalis*	63.213		65.326		
*Z. cucurbitae*	79.797		51.782		
*B. oleae*	14.186		66.527		
*C. capitata*	83.920		65.283		
*C. quilicii*	82.305		69.623		

***p < 0.001, **p < 0.01, n.s. p > 0.05.

**FIGURE 1:**
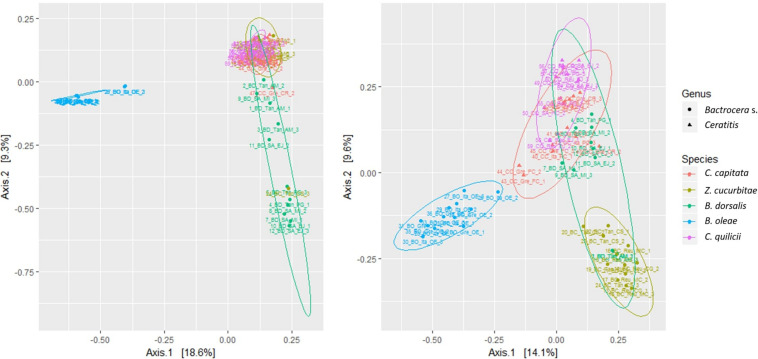
Multivariate ordination (PCOA) of gut microbial assemblages in five target fruit fly species (*B. dorsalis, Z. cucurbitae, B. oleae, C. capitata, C. quilicii;* dataset A); Shape = Fruit fly genus, Color = Fruit fly species; Left: Abundance data; Right: Presence-absence data.

The PCOAs of the five species included in the balanced experiments (Dataset A, Figure 1) only accounted for a relatively limited amount of variation, explaining in total 27.9 and 23.7% of variability (PC1 + PC2, 4th root transformed data and presence-absence, respectively). The 95% confidence ellipses allowed resolving *B. oleae* from all other species. Adding the additional species to the PCOA (data not shown), allowed accounting for 25.0 and 18.8% of variability (PC1 + PC2, 4th root transformed data and presence-absence, respectively). Again, inspection of the 95% confidence ellipses showed that only *B. oleae* clustered separately from all other species. Even when removing *B. oleae* from the PCOA an extensive overlap between the different species was still observed. The preliminary analysis of separate PCoAs for each of the fruit fly species targeted in this study did not provide additional suggestions on possible patterns related to location or host-plant (data not shown).

**FIGURE 2:**
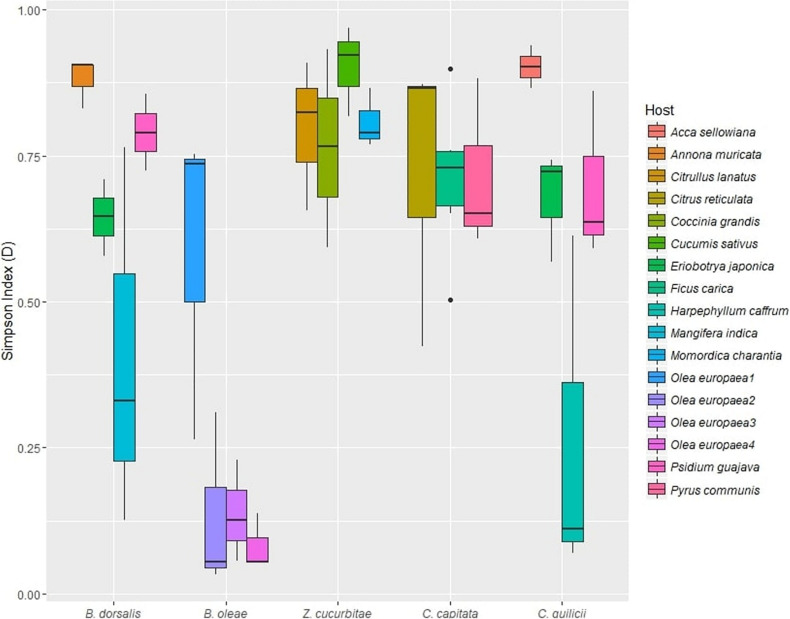
Alpha diversity (Simpson index, D) of gut microbial assemblages in five target fruit fly species (*B. dorsalis, Z. cucurbitae, B. oleae, C. capitata, C. quilicii*; dataset B) sampled in two locations, from two host plants at each location.

The mean alpha diversity, as estimated by the Simpson index, across all samples (Dataset A) was *D* = 0.62 (median: 0.72, SD: 0.28). ANOVA revealed a significant effect of both fruit fly species (*p* < 0.05) and host plant (*p* < 0.01) on gut microbiome diversity (Table 3, Supplementary Table S10, and Figure 2). *Post hoc* tests revealed significantly lower alpha diversity in *B. oleae* (*D* = 0.234; SD: 0.27) (Supplementary Table S10) compared to all other species except *C. capitata*. Effects of host plants on gut microbiome diversity were found for *B. oleae* between two varieties of *Olea europaea* and for *C. quilicii* between *Harpephyllum caffrum* and *Eriobotrya japonica* (Supplementary Table S10).

**TABLE 3:** ANOVA testing differences in alpha diversity (as estimated by the Simpson index, D; dataset A) of microbiome profiles of five fruit fly species (FFSp, *B. dorsalis, Z. cucurbitae, B. oleae, C. capitata, C. quilicii)* sampled in two locations [Lo(FFSp)] from two host plants within each location [Ho(FFSpxLo)].

	*df*	Mean Sq	*F*-value	*p*	
**Abundance-data**					
FFSp	4	0.602	5.651	0.043	*
Lo(FFSp)	5	0.107	1.008	0.461	n.s.
Ho(FFSpxLo)	10	0.106	4.072	0.001	***
Residual	40	0.026			

***p < 0.001, *p < 0.05, n.s.p > 0.05.

**FIGURE 3:**
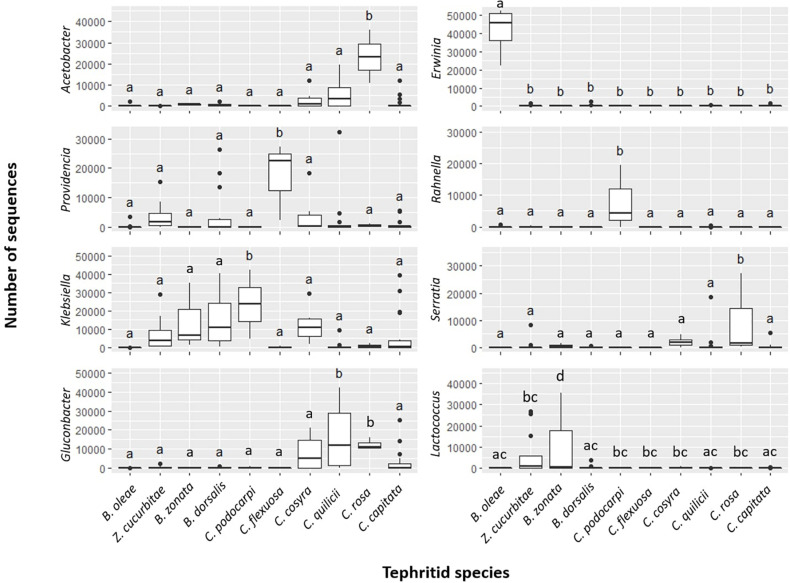
Abundances (as estimated in number of reads; dataset B) of most representative bacterial genera in ten targeted fruit fly species. Results are reported for bacterial genera producing significant differences in at least 8 pairwise tests out of 9 (FDR-corrected *p* < 0.01) and contributing to > 5% of dissimilarity between groups. For each species, significance letters for pairwise tests are indicated (see also Table 4 and Supplementary Table 12).

The permutational similarity percentage (SIMPER) analysis (Clarke, 1993) (Dataset B, Table 4, Figure 3, and Supplementary Table S12) suggested that five of the 10 investigated fruit fly species had characteristic associations with one or more bacterial genera. These putative associations were observed in all (9 out of 9) pairwise comparisons involving (a) *C. flexuosa*, which showed comparably higher abundances of reads from the genus *Providencia* (average abundance = 31.73%, *SD* = 24.45%) (b) *C. podocarpi*, with higher abundances of *Klebsiella* (average abundance = 52.83%, *SD* = 62.55%) and *Rahnella* (average abundance = 17.70%, *SD* = 25.03%)*;* (c) *C. rosa* with higher abundances of *Acetobacter* (average abundance = 55.30%, *SD* = 11.45%) and *Serratia* (average abundance = 0.06%, *SD* = 0.10%); (d) *B. oleae*, with higher abundances of *Erwinia* (average abundance = 93.28%, *SD* = 19.98%) and (e) *B. zonata*, with significantly higher abundances of *Lactococcus* (average abundance = 22.63%, *SD* = 38.71%). Other bacterial genera significantly contributed to the dissimilarity in most of the pairwise comparisons, such as *Morganella* and *Pantoea* in *C. capitata*, *Enterobacter* in *B. dorsalis* and *Gluconobacter* in *C. quilicii.* In *C. cosyra* none of the bacterial genera significantly contributed to more of 5% to the dissimilarity in at least five out of nine pairwise tests.

**TABLE 4:** Pairwise SIMPER permutational tests (10,000 iterations) between fruit fly species (*C. capitata, C. flexuosa, C. podocarpi, C. quilicii, C. rosa, C. cosyra, B. dorsalis, B. oleae, B. zonata, Z. cucurbitae*; dataset B).

	Bacterial genera significantly contributing to > 5% dissimilarity	*C. capitata*	*C. flexuosa*	*C. podocarpi*	*C. quilicii*	*C. rosa*	*C. cosyra*	*B. dorsalis*	*B. oleae*	*B. zonata*	*Z. cucurbitae*	Proportion of significant pairwise tests (FDR *p* < 0.05)
*C. capitata*	*Morganella*		*		*		*	*	*		*	6/9
	*Pantoea*				*		*	*	*		*	5/9
*C. flexuosa*	*Providencia*	*		*	*	*	*	*	*	*	*	9/9
*C. podocarpi*	*Klebsiella*	*	*		*	*	*	*	*	*	*	9/9
	*Rahnella*	*	*		*	*	*	*	*	*	*	9/9
*C. quilicii*	*Gluconobacter*	*	*	*			*	*	*	*	*	8/9
*C. rosa*	*Acetobacter*	*	*	*	*		*	*	*	*	*	9/9
	*Serratia*	*	*	*	*		*	*	*	*	*	9/9
*C. cosyra*	−											
*B. dorsalis*	*Enterobacter*	*	*		*	*	*		*		*	7/9
*B. oleae*	*Erwinia*	*	*	*	*	*	*	*		*	*	9/9
*B. zonata*	*Lactococcus*	*	*	*	*	*	*	*	*		*	9/9
*Z. cucurbitae*	*Lactococcus*	*			*			*	*	*		5/9
	*Ochrobactrum*	*			*		*	*	*			5/9

Results are reported for bacterial genera producing significant differences in at least 5 pairwise tests out of 9 (*: FDR-corrected p < 0.01) and contributing to > 5% of dissimilarity between groups. The complete results are available in Supplementary Table 13.

## Discussion

One of the main difficulties in the analysis of relationships between gut microbiome profiles and life history traits, including host plant choice, is represented by the high intra- and -interspecific variability of gut microbiomes that include thousands of ASVs (e.g., see De Cock et al., 2019). Differences between microbiome profiles can be related to life history traits and environmental factors including life stage (Lauzon et al., 2009; Andongma et al., 2015), diet (Santo Domingo et al., 1998; De Vries et al., 2004; Colman et al., 2012; Morrow et al., 2015), or technical artifacts (De Cock et al., 2019). Consistent with what has been reported in other studies on frugivorous tephritids (Thaochan et al., 2010; Wang et al., 2011; Andongma et al., 2015; Morrow et al., 2015; Augustinos et al., 2019), the gut microbiome profiles of third instar larvae of the ten fruit fly species targeted by the present study were mainly composed of Proteobacteria and Firmicutes which together represented more than 98.49% of reads in all tephritid species targeted. Andongma et al. (2015) suggested that Proteobacteria might be the most abundant phylum in earlier developmental stages of *Bactrocera*, while Firmicutes the most abundant in adult stages, possibly as a result of changes in habitat and diet. The dominance of Proteobacteria in larval stages is consistent with what is observed in the present work not only for *Bactrocera* and *Zeugodacus* but also for *Ceratitis* and it further confirms variation in microbiome profiles across developmental stages, as also described in *C. capitata* (De Cock et al., 2019).

Previous studies reported contrasting results on the most abundant gut bacterial families in tephritid fruit flies. While most of these studies report Enterobacteriaceae (Proteobacteria) as a major, dominant component of fruit fly gut microbiomes (Kuzina et al., 2001; Behar et al., 2008b; Wang et al., 2011; Wang H. et al., 2014) there are also notable exceptions such in Andongma et al. (2015) where Comamonadaceae are shown to represent a dominant taxon in immature stages of *B. dorsalis*. The dominance of Enterobacteriaceae, as the major component of the gut microbiome of most of the targeted species was confirmed by the results of the present study, with the notable exception of *C. quilicii* and *C. rosa* for which Acetobacteraceae (Proteobacteria) was the bacterial family with the highest abundance. At genus level and ASV level, we observed a high variability both between fruit fly species and within species. Only a few bacterial genera (*Klebsiella*, *Enterobacter*, and *Bacillus*) were present in a large proportion of samples, albeit with high variability in their relative abundance. Patterns observed for *C. capitata* were generally in line with what previously observed in laboratory populations (De Cock et al., 2019), with Proteobacteria and Firmicutes as the most abundant phyla and Enterobacteriaceae representing the most abundant family.

Regardless the relatively high number of individual guts used for this screening, the microbiome profiles of larvae collected in the field from their natural host plants showed highly variable patterns both between and within species, with intraspecific variation often, but not always, showing significant changes according to the host plant attacked. Interspecific variation of microbiome profiles was significantly affected by larval diet only in the two most polyphagous fruit fly species, *B. dorsalis* and *C. capitata*. Similarly, both the polyphagous *C. quilicii* and the monophagous *B. oleae* seemed also affected by host plant, even if to a lesser extent (i.e., they showed differences in univariate patterns of diversity but not in their multivariate patterns) while the oligophagous *Z. cucurbitae* did not seem significantly affected by host-plant choice. Regardless of that, the geographic variability of microbiome profiles from fruit fly populations thousands of km distant was relatively limited (relatively, as significant effects could only be observed from the analysis of presence/absence data). This suggests that the variable patterns observed across fruit fly species and host plants (particularly for *B. dorsalis* and *C. capitata*), are geographically consistent, even at large spatial scales (i.e., across different countries in the same continent). More focused experimental designs (i.e., based on a larger number of replicated samples and hosts) are now needed for a more detailed characterization of changes in the microbiome profiles of *B. dorsalis* and *C. capitata* across different host fruits. It would also be of interest to include the microbiome of the fruit that the larvae are sampled from in these studies.

Similarly, we did not find indications of obvious relationships either between microbiome profile diversity and fruit fly dietary breadth or between the microbiome profiles of the three different genera targeted in this study (*Ceratitis* on one hand and the closely related *Bactrocera/Zeugodacus* on the other).

From our observation, a core microbiome for the targeted fruit fly species could be defined only at family level, where the family Enterobacteriaceae was the single recurrent element in all samples. At genus or ASV level, however, we could not identify universal core microbiome elements shared by all fruit fly species tested. For individual fruit fly species, however, we could identify a set of key bacterial genera whose abundance was significantly higher in particular fruit fly species, irrespective of the host plant or sampling location considered. Major differences (i.e., significantly higher in all pairwise comparisons implemented) were found in the abundance patterns of seven bacterial genera in five fruit fly species considered. These were *Erwinia* in *B. oleae*, *Lactococcus* in *B. zonata*, *Providencia* in *C. flexuosa*, *Klebsiella*, and *Rahnella* in *C. podocarpi* and *Acetobacter* and *Serratia* in *C. rosa*. Other but less pronounced differences (as significant in a large proportion of pairwise comparison but not in all) were found for genera such as *Ochrobactrum* in *Z. cucurbitae*, *Gluconobacter* in *C. quilicii* and *Enterobacter* in *B. dorsalis*. Further experimental validation is now needed to verify the generality of these patterns and to test the occurrence of stable associations between larval dietary niche and the presence of the above-mentioned gut symbionts.

In herbivorous insects, gut microbes can aid with the breakdown of complex polysaccharides that make up the plant cell wall, or supplement the nutritionally poor plant diet with nitrogen, vitamins and sterols (Douglas, 2009; Ben-Yosef et al., 2010, 2014). There is also evidence that they take part in the detoxification of plant allelochemicals (Hammer and Bowers, 2015). The relationship between the genus *Erwinia* and *B. oleae* has been studied extensively as it is a prime example of the coevolution between an insect and its gut microbiome (Capuzzo et al., 2005; Ben-Yosef et al., 2014, 2015; Estes et al., 2014; Blow et al., 2016; Pavlidi et al., 2017). It is hypothesized that the close relationship with this bacterium allows *B. oleae* to exploit olives as a food source by detoxifying plant defense compounds (Ben-Yosef et al., 2015) and providing additional nutrition (Ben-Yosef et al., 2014). “*Candidatus* Erwinia dacicola” allows larvae of *B. oleae* to develop in unripe olives, which contain high concentrations of the toxin oleuropein (Ben-Yosef et al., 2015; Pavlidi et al., 2017). As such, host-associated microbial communities seem to play an important role in the evolution and possibly speciation of the host (Zilber-Rosenberg and Rosenberg, 2008), in particular in fruit flies (Behar et al., 2008a; Ben-Yosef et al., 2010, 2014, 2015).

The bacterial genus *Ochrobactrum*, which in our study showed higher abundance in *Z. cucurbitae* in a number of interspecific pairwise comparisons, has often been reported as a plant endophyte of Cucurbitaceae (Weller et al., 2006; Akbaba and Ozaktan, 2018) and described in a number of cucurbit feeder fruit flies including *Z. cucurbitae* (Mishra and Sharma, 2018), *Z. tau* (pumpkin fly) (Khan et al., 2014; Prabhakar et al., 2013; Luo et al., 2018), and in the polyphagous *B. tryoni* (Jessup and Mccarthy, 1993). Similarly, the bacterial genus *Rahnella* which we consistently found in higher abundances in the gut microbiome of *C. podocarpi* has been reported in different species of bark beetles (Lacey et al., 2007; Vasanthakumar et al., 2009; Brady et al., 2014; Hernández-García et al., 2017), many of which feed on bark of coniferous trees. While these beetles and *C. podocarpi* do not share a taxonomic link, they do share a similar host: *C. podocarpi* exclusively targets members of the family Podocarpaceae, which also belong to the group of conifer trees. Even more so, the fruits of *Afrocarpus falcatus* (syn. *Podocarpus falcatus*) are known to be edible, but very resinous (source ICRAF Agroforestree Database; Oduol et al., 1988). The presence of *Rahnella* in the gut of these insects could be linked to the presence of this resin, which is also found plentiful in other coniferous trees.

## Conclusion

Consistent with literature, we found that the gut microbiome of all fruit fly species included in the present study, was composed mainly of members of the bacterial phyla Proteobacteria and Firmicutes. At family level, we found that the family of Enterobacteriaceae was the dominant component in most species, except in *C. quilicii* and *C. rosa* where Acetobacteraceae was the dominant bacterial family. Despite heterogeneous abundances, we consistently observed Enterobacteriaceae across all samples, making it the single bacterial family that could be considered a part of the “core” gut microbiome. At genus level and ASV level, we observed a high variability both between fruit fly species (regardless of fruit fly genus) and within species. As such, we could not identify “core” gut microbiome members at genus or ASV levels that were shared across the targeted fruit flies. The few bacterial genera (*Klebsiella*, *Enterobacter* and *Bacillus*) that were present in most samples, showed a high variability in their relative abundance. Interestingly, we observed that interspecific variation of microbiome profiles was significantly affected by larval diet only in a part of the targeted fruit fly species (i.e., the most polyphagous ones, *B. dorsalis* and *C. capitata*), and that the observed patterns were geographically consistent. Finally, we could identify a number of bacterial genera (such as *Erwinia*, *Ochrobactrum* and *Rahnella*) that were consistently associated with particular fruit fly species (respectively *B. oleae*, *Z. cucurbitae*, and *C. podocarpi*). With these results, the present study provides a first comparative analysis of the gut microbiome of major fruit fly pests as well as, new base line information for future studies that will further investigate the functional role of the described associations.

## Data Availability Statement

The datasets presented in this study can be found in online repositories. The names of the repository/repositories and accession number(s) can be found at: https://www.ncbi.nlm.nih.gov/, SRR8741994 to SRR8742034.

## Author Contributions

MDM, AW, MV, KB, and PV designed the research and secured the funding. MDC and MV designed and performed the experiments. MDC and MV analyzed the data with input from all other authors. MDC drafted the manuscript. All authors proofread, edited, and approved the manuscript.

## Conflict of Interest

The authors declare that the research was conducted in the absence of any commercial or financial relationships that could be construed as a potential conflict of interest.

## Footnotes

1 http://bccm.belspo.be/

## Supplementary Material

FIGURE S1 Bubble plot representing the bacterial phylum composition per sample. Bubble color: number of bacterial genera per phylum; Bubble size: Log of reads per phylum; X-axis: Sample labels build with a consistent structure: XX_YY_ZZ in which XX is fruit fly species, YY is sample location and ZZ is host plant.

FIGURE S2 Relative abundances (%, as estimated from number of reads) of the dominant bacterial families per fruit fly species. Error bars (SD) as calculated from averaged three replicates per species are indicated.

TABLE S1 Overview of sample design implemented in this study. Blue: balanced experiment including five species (*B. dorsalis, Z. cucurbitae, B. oleae, C. capitata, C. quilicii*) sampled in two locations from two host plants within each location (three replicate samples for each host plant species); Gray: additional samples complementing the balanced experiment and including additional fruit fly species (and including *B. zonata, C. cosyra, C. rosa, C. flexuosa, C. podocarpi*), locations and host plants. Blue = dataset A; Blue + Gray = dataset B.

TABLE S2 Collection data of samples considered in this study.

TABLE S3 Detailed list ofthe strain composition of the MOCK community.

TABLE S4 Detailed analytical pipeline implemented for genomic data filtering and taxon assignment.

TABLE S5 Identification and number of reads for ASVs that were detected in the negative control.

TABLE S6 *A posteriori* pairwise comparisons (permutational t-statistics) for the significant effects detected by the PERMANOVA test reported in Table 1 (fourth root transformed data; dataset A). ‘***’ = *p* < 0.001, ‘**’ = *p* < 0.01, ‘**’ = *p* < 0.05, ‘n.s.’ = *p* > 0.05.

TABLE S7 *A posteriori* pairwise comparisons (permutational t-statistics) for the significant effects detected by the PERMANOVA test reported in Table 1 (presence-absence data; dataset A). ‘***’ = *p* < 0.001, ‘**’ = *p* < 0.01 ‘*’ = *p* < 0.05, ‘n.s.’ = *p* > 0.05.

TABLE S8 *A posteriori* pairwise comparisons (permutational t-statistics) for the significant effects detected by the PERMDISP test reported in Table 2 (fourth root transformed data; dataset A). ‘***’ = *p* < 0.001, ‘**’ = *p* < 0.01, ‘*’ = *p* < 0.05, ‘n.s.’ = *p* > 0.05.

TABLE S9 *A posteriori* pairwise comparisons (permutational t-statistics) for the significant effects detected by the PERMDISP test reported in Table 2 (presence-absence data; dataset A). ‘***’ = *p* < 0.001, ‘**’ = *p* < 0.01, ‘*’ = *p* < 0.05, ‘n.s.’ = *p* > 0.05.

TABLE S10 *A posteriori* pairwise comparisons (Student-Newman-Keuls test) for the significant effects detected by the ANOVA test reported in Table 3. ‘***’ = *p* < 0.001, ‘**’ = *p* < 0.01, ‘*’ = *p* < 0.05, ‘n.s.’ = *p* > 0.05 (Dataset A).

TABLE S11 Most abundant bacterial genera observed in each fruit fly species (% as estimated from number of reads).

TABLE S12 Permutational SIMPER analysis (10,000 iterations; dataset B) of the microbiome assemblages of 10 fruit fly species (*C. capitata, C. flexuosa, C. podocarpi, C. quilicii, C. rosa, C.cosyra, B. dorsalis, B. oleae, B. zonata, Z. cucurbitae*). sd: standard deviation of contribution to dissimilarity between groups; ratio: average to sd ratio; FDR p: probability value after False Discovery Rate correction (Benjamini and Hochberg, 1995); *: FDR p < 0.05, **: FDR p < 0.01. Results are reported for bacterial genera contributing up to 95% of dissimilarity between fruit fly species. Bacterial genera with contribution to average group dissimilarity >5% and significant FDR p are highlighted in blue.
