# Supplementary figures and images for: Comparative Microbiomics of Tephritid Frugivorous Pests (Diptera: Tephritidae) From the Field: A Tale of High Variability Across and Within Species
Source: Front Microbiol. 2020 Aug 11;11:1890. doi: 10.3389/fmicb.2020.01890 (PMC7431611; doi:10.3389/fmicb.2020.01890)

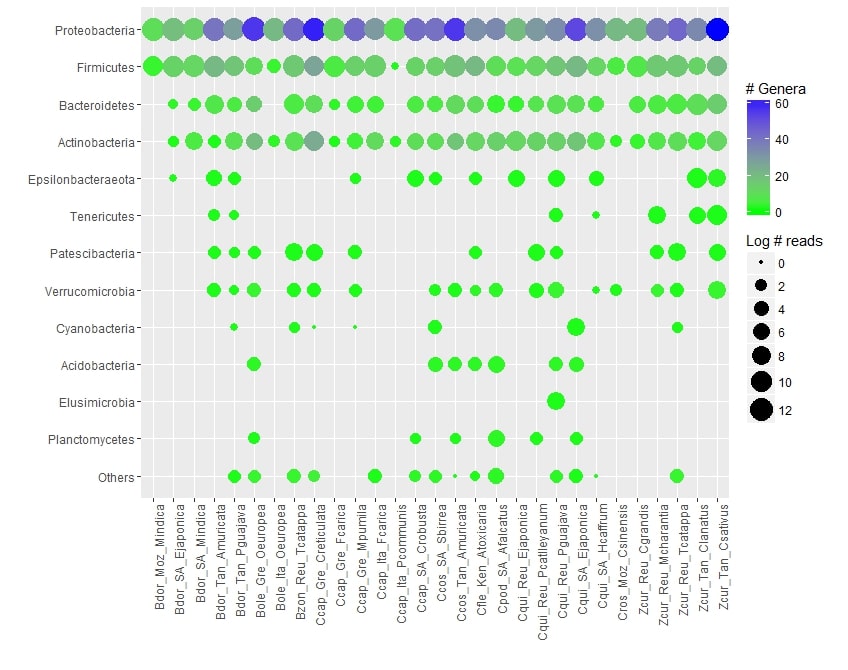

Supplement: FIGURE S1 — Bubble plot representing the bacterial phylum composition per sample. Bubble color: number of bacterial genera per phylum; Bubble size: Log of reads per phylum; X-axis: Sample labels build with a consistent structure: XX_YY_ZZ in which XX is fruit fly species, YY is sample location and ZZ is host plant. [file Image_1.JPEG]

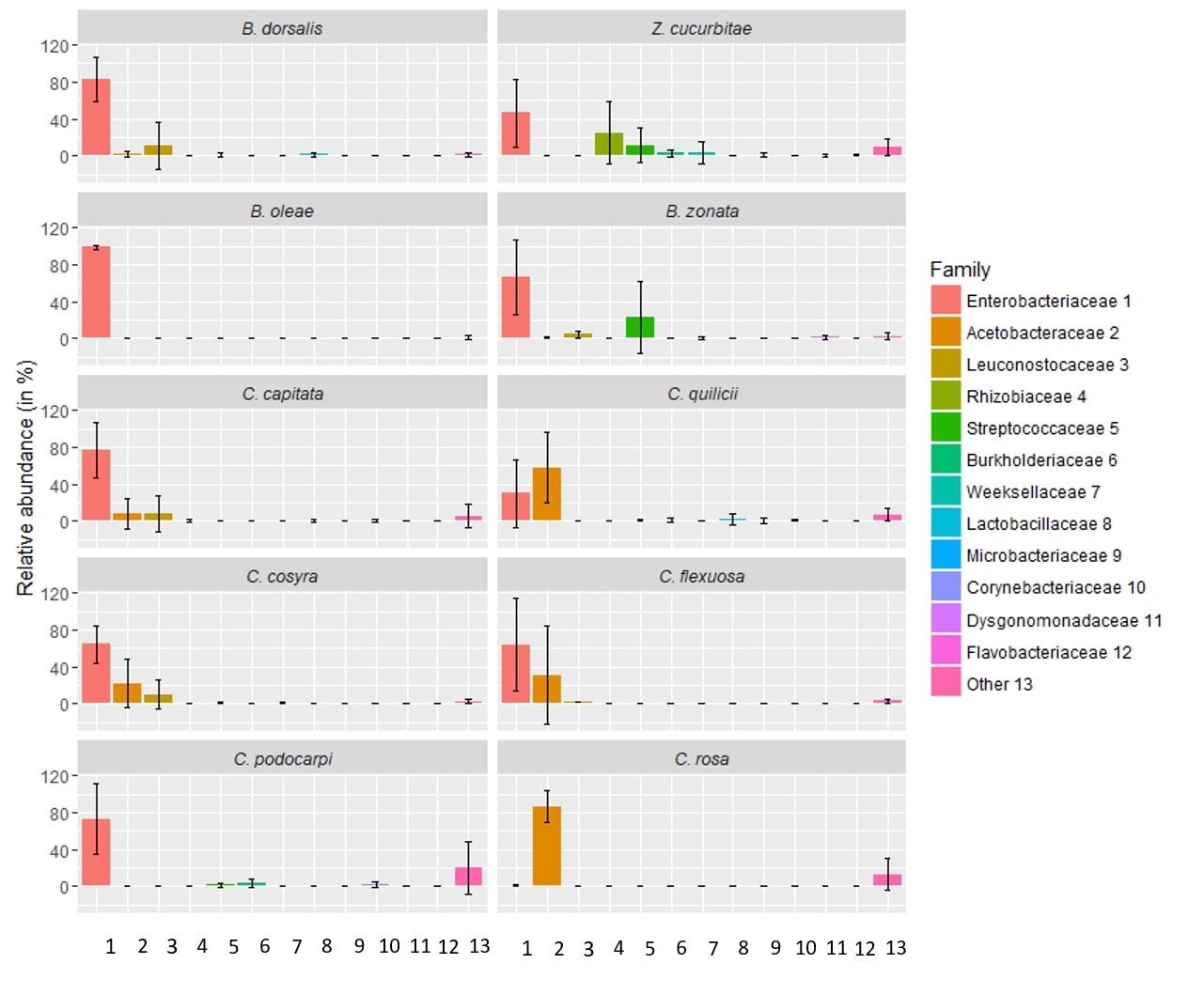

Supplement: FIGURE S2 — Relative abundances (%, as estimated from number of reads) of the dominant bacterial families per fruit fly species. Error bars (SD) as calculated from averaged three replicates per species are indicated. [file Image_2.JPEG]
