# Supplementary material for: Comparative Microbiomics of Tephritid Frugivorous Pests (Diptera: Tephritidae) From the Field: A Tale of High Variability Across and Within Species
Source: Front Microbiol. 2020 Aug 11;11:1890. doi: 10.3389/fmicb.2020.01890 (PMC7431611; doi:10.3389/fmicb.2020.01890)
Supplement: TABLE S1 — Overview of sample design implemented in this study. Blue: balanced experiment including five species (B. dorsalis, Z. cucurbitae, B. oleae, C. capitata, C. quilicii) sampled in two locations from two host plants within each location (three replicate samples for each host plant species); Gray: additional samples complementing the balanced experiment and including additional fruit fly species (and including B. zonata, C. cosyra, C. rosa, C. flexuosa, C. podocarpi), locations and host plants. Blue = dataset A; Blue + Gray = dataset B. [file Table_1.DOCX]

Supplementary Table S1 : Overview of sample design implemented in this study. Blue: balanced experiment including five species (*B. dorsalis, Z. cucurbitae, B. oleae, C. capitata, C. quilicii*) sampled in two locations from two host plants within each location (three replicate samples for each host plant species); Grey: additional samples complementing the balanced experiment and including additional fruit fly species (and including *B. zonata, C. cosyra, C. rosa, C. flexuosa, C. podocarpi*), locations and host plants. Blue = dataset A; Blue + Grey = dataset B.
