# Supplementary material for: Comparative Microbiomics of Tephritid Frugivorous Pests (Diptera: Tephritidae) From the Field: A Tale of High Variability Across and Within Species
Source: Front Microbiol. 2020 Aug 11;11:1890. doi: 10.3389/fmicb.2020.01890 (PMC7431611; doi:10.3389/fmicb.2020.01890)
Supplement: TABLE S2 — Collection data of samples considered in this study. [file Table_2.DOCX]

Supplementary Table S2 : Collection data of samples considered in this study

| Genus | Species | Location | Host plant | Collection data | Collection by |
| --- | --- | --- | --- | --- | --- |
| *Zeugodacus* | *Z. cucurbitae* | Reunion | *Coccinia grandis* | 11/08/2016 | H. Delatte |
| *Zeugodacus* | *Z. cucurbitae* | Reunion | *Momordica charantia* | 26/08/2016 | H. Delatte |
| *Zeugodacus* | *Z. cucurbitae* | Tanzania | *Citrullus lanatus* | 21/08/2017 | J. Kusolwa |
| *Bactrocera* | *Z. cucurbitae* | Tanzania | *Cucumis sativus* | 16/08/2017 | J. Kusolwa |
| *Bactrocera* | *B. dorsalis* | Mozambique | *Mangifera indica* | 5/01/2017 | L. Bota |
| *Bactrocera* | *B. dorsalis* | South Africa | *Eriobotrya japonica* | 27/09/2016 | A. Manrakhan |
| *Bactrocera* | *B. dorsalis* | South Africa | *Mangifera indica* | 12/02/2017 | A. Manrakhan |
| *Bactrocera* | *B. dorsalis* | Tanzania | *Annona muricata* | 29/07/2017 | M. De Cock |
| *Bactrocera* | *B. dorsalis* | Tanzania | *Psidium guajava* | 19/08/2017 | J. Kusolwa |
| *Bactrocera* | *B. oleae* | Greece | *Olea europea1* | 6/12/2017 | M. De Cock |
| *Bactrocera* | *B. oleae* | Greece | *Olea europea2* | 8/12/2017 | M. De Cock |
| *Bactrocera* | *B. oleae* | Greece | *Olea europea3* | 25/10/2016 | N. Papadopoulos |
| *Bactrocera* | *B. oleae* | Italy | *Olea europea1* | 8/11/2016 | M. Virgilio |
| *Bactrocera* | *B. oleae* | Italy | *Olea europea2* | 8/11/2016 | M. Virgilio |
| *Bactrocera* | *B. zonata* | Reunion | *Terminalia catappa* | 19/08/2016 | H. Delatte |
| *Ceratitis* | *C. capitata* | Greece | *Citrus reticulata* | 5/11/2016 | N. Papadopoulos |
| *Ceratitis* | *C. capitata* | Greece | *Ficus carica* | 1/10/2017 | N. Papadopoulos |
| *Ceratitis* | *C. capitata* | Greece | *Malus pumila* | 10/09/2016 | N. Papadopoulos |
| *Ceratitis* | *C. capitata* | Italy | *Ficus carica* | -/8/2016 | M. Virgilio |
| *Ceratitis* | *C. capitata* | Italy | *Pyrus communis* | 4/8/2016 | M. Virgilio |
| *Ceratitis* | *C. capitata* | South Africa | *Coffea robusta* | 23/08/2016 | A. Manrakhan |
| *Ceratitis* | *C. cosyra* | South Africa | *Sclerocarya birrea* | 7/02/2017 | A. Manrakhan |
| *Ceratitis* | *C. cosyra* | Tanzania | *Annona muricata* | 22/08/2017 | M. De Cock |
| *Ceratitis* | *C. flexuosa* | Kenya | *Antiaris toxicaria* | 26/12/2016 | W. Okeka |
| *Ceratitis* | *C. podocarpi* | South Africa | *Afrocarpus falcatus* | 19/06/2017 | A. Manrakhan |
| *Ceratitis* | *C. quilicii* | Reunion | *Eriobotrya japonica* | 11/08/2016 | H. Delatte |
| *Ceratitis* | *C. quilicii* | Reunion | *Psidium catlleyanum* | 11/08/2016 | H. Delatte |
| *Ceratitis* | *C. quilicii* | Reunion | *Psidium guajava* | 27/07/2016 | H. Delatte |
| *Ceratitis* | *C. quilicii* | South Africa | *Eriobotrya japonica* | 27/09/2016 | C. Weldon |
| *Ceratitis* | *C. quilicii* | South Africa | *Harpephyllum caffrum* | 29/01/2017 | C. Weldon |
| *Ceratitis* | *C. rosa* | Mozambique | *Citrus sinensis* | -/7/2016 | L. Bota |
