# Supplementary material for: Comparative Microbiomics of Tephritid Frugivorous Pests (Diptera: Tephritidae) From the Field: A Tale of High Variability Across and Within Species
Source: Front Microbiol. 2020 Aug 11;11:1890. doi: 10.3389/fmicb.2020.01890 (PMC7431611; doi:10.3389/fmicb.2020.01890)
Supplement: TABLE S5 — Identification and number of reads for ASVs that were detected in the negative control. [file Table_5.DOCX]

Supplementary Table S5: Identification and number of reads for ASVs that were detected in the negative control.

| Kingdom | Phylum | Family | Genus | Species | Total # of reads | # in Blank |
| --- | --- | --- | --- | --- | --- | --- |
| Bacteria | Proteobacteria | Enterobacteriaceae | Escherichia | NA | **7439** | **774** |
| Bacteria | Firmicutes | Staphylococcaceae | Staphylococcus | NA | **2209** | **55** |
| Bacteria | Proteobacteria | Enterobacteriaceae | Providencia | NA | **48** | **48** |
| Bacteria | Proteobacteria | Pseudomonadaceae | Pseudomonas | NA | **43** | **38** |
| Bacteria | Proteobacteria | Moraxellaceae | Acinetobacter | NA | **29** | **29** |
| Bacteria | Actinobacteria | Micrococcaceae | Arthrobacter | NA | **25** | **25** |
| Bacteria | Proteobacteria | Burkholderiaceae | Curvibacter | gracilis | **3195** | **19** |
| Bacteria | Proteobacteria | Moraxellaceae | Acinetobacter | NA | **17** | **17** |
| Bacteria | Proteobacteria | Burkholderiaceae | Pelomonas | NA | **1371** | **15** |
| Bacteria | Firmicutes | Family_XI | Anaerococcus | NA | **13** | **13** |
| Bacteria | Proteobacteria | Caulobacteraceae | Brevundimonas | NA | **41** | **12** |
