# Supplementary material for: Comparative Microbiomics of Tephritid Frugivorous Pests (Diptera: Tephritidae) From the Field: A Tale of High Variability Across and Within Species
Source: Front Microbiol. 2020 Aug 11;11:1890. doi: 10.3389/fmicb.2020.01890 (PMC7431611; doi:10.3389/fmicb.2020.01890)
Supplement: TABLE S6 — A posteriori pairwise comparisons (permutational t-statistics) for the significant effects detected by the PERMANOVA test reported in Table 1 (fourth root transformed data; dataset A). ‘***’ = p < 0.001, ‘**’ = p < 0.01, ‘**’ = p < 0.05, ‘n.s.’ = p > 0.05. [file Table_6.DOCX]

Supplementary Table S6 : A posteriori pairwise comparisons (permutational t-statistics) for the significant effects detected by the PERMANOVA test reported in Table 1 (fourth root transformed data). ‘***’ = p < 0.001, ‘**’ = p < 0.01, ‘*’ = p < 0.05, ‘n.s.’ = p > 0.05

| **Fruit fly Species** |  |  |  |
| --- | --- | --- | --- |
| Groups | t | p-value |  |
| *B. dorsalis - Z. cucurbitae* | 1.738 | 0.049 | * |
| *B. dorsalis - B. oleae* | 3.115 | 0.008 | ** |
| *B. dorsalis - C. capitata* | 1.430 | 0.101 | n.s. |
| *B. dorsalis - C. quilicii* | 1.653 | 0.049 | * |
| *Z. cucurbitae - B. oleae* | 3.381 | 0.008 | ** |
| *Z. cucurbitae - C. capitata* | 1.547 | 0.068 | n.s. |
| *Z. cucurbitae - C. quilicii* | 1.708 | 0.049 | * |
| *B. oleae - C. capitata* | 2.549 | 0.011 | * |
| *B. oleae - C. quilicii* | 2.731 | 0.011 | * |
| *C. capitata - C. quilicii* | 1.229 | 0.198 | n.s. |
|  |  |  |  |
| **Plant host** |  |  |  |
| Groups | t | p-value |  |
| ***B. dorsalis*** |  |  |  |
| *A. muricata - P. guajava* | 3.073 | 0.021 | * |
| *A. muricata - M. indica* | 2.572 | 0.023 | * |
| *A. muricata - E. japonica* | 3.162 | 0.021 | * |
| *P. guajava - M. indica* | 2.065 | 0.040 | * |
| *P. guajava - E. japonica* | 2.451 | 0.029 | * |
| *M. indica - E. japonica* | 1.440 | 0.156 | n.s. |
|  |  |  |  |
| ***Z. cucurbitae*** |  |  |  |
| *C. grandis - M. charantia* | 1.386 | 0.182 | n.s. |
| *C. grandis - C. lanatus* | 1.911 | 0.182 | n.s. |
| *C. grandis - C. sativus* | 1.657 | 0.182 | n.s. |
| *M. charantia - C. lanatus* | 1.512 | 0.182 | n.s. |
| *M. charantia - C. sativus* | 1.429 | 0.182 | n.s. |
| *C. lanatus - C. sativus* | 1.315 | 0.192 | n.s. |
|  |  |  |  |
| ***B. oleae*** |  |  |  |
| *O. europea1 - O. europea2* | 1.446 | 0.325 | n.s. |
| *O. europea1 - O. europea3* | 1.574 | 0.325 | n.s. |
| *O. europea1 - O. europea4* | 1.766 | 0.325 | n.s. |
| *O. europea2 - O. europea3* | 0.699 | 0.628 | n.s. |
| *O. europea2 - O. europea4* | 1.147 | 0.471 | n.s. |
| *O. europea3 - O. europea4* | 0.751 | 0.628 | n.s. |
|  |  |  |  |
| ***C. capitata*** |  |  |  |
| *F. carica1 - P. communis* | 2.022 | 0.049 | * |
| *F. carica1 - F. carica2* | 2.309 | 0.037 | * |
| *F. carica1 - C. reticulata* | 1.614 | 0.071 | n.s. |
| *P. communis - F. carica2* | 4.442 | 0.012 | * |
| *P. communis - C. reticulata* | 2.175 | 0.037 | * |
| *F. carica2 - C. reticulata* | 2.374 | 0.037 | * |
|  |  |  |  |
| ***C. quilicii*** |  |  |  |
| *H. caffrum - E. japonica1* | 1.950 | 0.100 | n.s. |
| *H. caffrum - P. guajava* | 1.785 | 0.100 | n.s. |
| *H. caffrum - E. japonica2* | 1.767 | 0.100 | n.s. |
| *E. japonica1 - P. guajava* | 1.590 | 0.100 | n.s. |
| *E. japonica1 - E. japonica2* | 1.506 | 0.100 | n.s. |
| *P. guajava - E. japonica2* | 1.549 | 0.100 | n.s. |
