# Supplementary material for: Comparative Microbiomics of Tephritid Frugivorous Pests (Diptera: Tephritidae) From the Field: A Tale of High Variability Across and Within Species
Source: Front Microbiol. 2020 Aug 11;11:1890. doi: 10.3389/fmicb.2020.01890 (PMC7431611; doi:10.3389/fmicb.2020.01890)
Supplement: TABLE S7 — A posteriori pairwise comparisons (permutational t-statistics) for the significant effects detected by the PERMANOVA test reported in Table 1 (presence-absence data; dataset A). ‘***’ = p < 0.001, ‘**’ = p < 0.01 ‘*’ = p < 0.05, ‘n.s.’ = p > 0.05. [file Table_7.DOCX]

Supplementary Table S7: *A posteriori* pairwise comparisons (permutational t-statistics) for the significant effects detected by the PERMANOVA test reported in Table 1 (presence-absence data). ‘***’ = p < 0.001, ‘**’ = p < 0.01, ‘*’ = p < 0.05, ‘n.s.’ = p > 0.05

| **Fruit fly Species** |  |  |  |
| --- | --- | --- | --- |
| Groups | t | p-value |  |
| *B. dorsalis - Z. cucurbitae* | 1.284 | 0.281 | n.s. |
| *B. dorsalis - B. oleae* | 1.836 | 0.121 | n.s. |
| *B. dorsalis - C. capitata* | 1.104 | 0.383 | n.s. |
| *B. dorsalis - C. quilicii* | 1.369 | 0.245 | n.s. |
| *Z. cucurbitae - B. oleae* | 2.255 | 0.074 | n.s. |
| *Z. cucurbitae - C. capitata* | 1.450 | 0.207 | n.s. |
| *Z. cucurbitae - C. quilicii* | 1.738 | 0.133 | n.s. |
| *B. oleae - C. capitata* | 1.790 | 0.129 | n.s. |
| *B. oleae - C. quilicii* | 2.224 | 0.076 | n.s. |
| *C. capitata - C. quilicii* | 1.250 | 0.297 | n.s. |
|  |  |  |  |
| **Location** |  |  |  |
| Groups | t | p-value |  |
| ***B. dorsalis*** |  |  |  |
| Tanzania - South Africa | 1.289 | 0.278 | n.s. |
|  |  |  |  |
| ***Z. cucurbitae*** |  |  |  |
| Reunion - Tanzania | 1.487 | 0.200 | n.s. |
|  |  |  |  |
| ***B. oleae*** |  |  |  |
| Italy - Greece | 1.163 | 0.349 | n.s. |
|  |  |  |  |
| ***C. capitata*** |  |  |  |
| Italy - Greece | 1.034 | 0.431 | n.s. |
|  |  |  |  |
| ***C. quilicii*** |  |  |  |
| South Africa - Reunion | 0.948 | 0.492 | n.s. |
|  |  |  |  |
| **Host plant** |  |  |  |
| Groups | t | p-value |  |
| ***B. dorsalis* - Tanzania** |  |  |  |
| *A. muricata - P. guajava* | 2.615 | 0.015 | * |
| ***B. dorsalis* - South Africa** |  |  |  |
| *M. indica - E. japonica* | 1.816 | 0.054 | n.s. |
|  |  |  |  |
| ***Z. cucurbitae* - Reunion** |  |  |  |
| *C. grandis - M. charantia* | 1.297 | 0.194 | n.s. |
| ***Z. cucurbitae* - Tanzania** |  |  |  |
| *C. lanatus - C. sativus* | 1.348 | 0.168 | n.s. |
|  |  |  |  |
| ***B. oleae* - Italy** |  |  |  |
| *O. europea1 - O. europea2* | 1.306 | 0.190 | n.s. |
| ***B. oleae* - Greece** |  |  |  |
| *O. europea3 - O. europea4* | 0.769 | 0.670 | n.s. |
|  |  |  |  |
| ***C. capitata* - Italy** |  |  |  |
| *F. carica1 - P. communis* | 2.401 | 0.016 | * |
| ***C. capitata* - Greece** |  |  |  |
| *F. carica2 - C. reticulata* | 2.275 | 0.020 | * |
|  |  |  |  |
| ***C. quilicii* - South Africa** |  |  |  |
| *H. caffrum - E. japonica1* | 1.584 | 0.080 | n.s. |
| ***C. quilicii* - Reunion** |  |  |  |
| *P. guajava - E. japonica2* | 1.406 | 0.138 | n.s. |
