# Supplementary material for: Comparative Microbiomics of Tephritid Frugivorous Pests (Diptera: Tephritidae) From the Field: A Tale of High Variability Across and Within Species
Source: Front Microbiol. 2020 Aug 11;11:1890. doi: 10.3389/fmicb.2020.01890 (PMC7431611; doi:10.3389/fmicb.2020.01890)
Supplement: TABLE S8 — A posteriori pairwise comparisons (permutational t-statistics) for the significant effects detected by the PERMDISP test reported in Table 2 (fourth root transformed data; dataset A). ‘***’ = p < 0.001, ‘**’ = p < 0.01, ‘*’ = p < 0.05, ‘n.s.’ = p > 0.05. [file Table_8.DOCX]

Supplementary Table S8: *A posteriori* pairwise comparisons (permutational t-statistics) for the significant effects detected by the PERMDISP test reported in Table 2 (fourth root transformed data). ‘***’ = p < 0.001, ‘**’ = p < 0.01, ‘*’ = p < 0.05, ‘n.s.’ = p > 0.05

| **Fruit fly Species** |  |  |  |
| --- | --- | --- | --- |
| Groups | t | p-value |  |
| *B. dorsalis - Z. cucurbitae* | 3.218 | 0.145 | n.s. |
| *B. dorsalis - B. oleae* | 1.860 | 0.191 | n.s. |
| *B. dorsalis - C. capitata* | 0.879 | 0.399 | n.s. |
| *B. dorsalis - C. quilicii* | 3.341 | 0.145 | n.s. |
| *Z. cucurbitae - B. oleae* | 5.720 | 0.145 | n.s. |
| *Z. cucurbitae - C. capitata* | 0.946 | 0.399 | n.s. |
| *Z. cucurbitae - C. quilicii* | 1.129 | 0.399 | n.s. |
| *B. oleae - C. capitata* | 2.158 | 0.172 | n.s. |
| *B. oleae - C. quilicii* | 5.202 | 0.145 | n.s. |
| *C. capitata - C. quilicii* | 1.470 | 0.329 | n.s. |
|  |  |  |  |
| **Host plant** |  |  |  |
| Groups | t | p-value |  |
| ***B. dorsalis*** |  |  |  |
| *A. muricata - P. guajava* | 0.984 | 0.599 | n.s. |
| *A. muricata - M. indica* | 3.291 | 0.201 | n.s. |
| *A. muricata - E. japonica* | 1.092 | 0.450 | n.s. |
| *P. guajava - M. indica* | 2.858 | 0.201 | n.s. |
| *P. guajava - E. japonica* | 0.651 | 0.901 | n.s. |
| *M. indica - E. japonica* | 3.974 | 0.201 | n.s. |
|  |  |  |  |
| ***Z. cucurbitae*** |  |  |  |
| *C. grandis - M. charantia* | 0.605 | 0.898 | n.s. |
| *C. grandis - C. lanatus* | 0.511 | 0.898 | n.s. |
| *C. grandis - C. sativus* | 0.951 | 0.898 | n.s. |
| *M. charantia - C. lanatus* | 0.892 | 0.898 | n.s. |
| *M. charantia - C. sativus* | 1.478 | 0.898 | n.s. |
| *C. lanatus - C. sativus* | 0.219 | 0.898 | n.s. |
|  |  |  |  |
| ***B. oleae*** |  |  |  |
| *O. europea1 - O. europea2* | 6.795 | 0.203 | n.s. |
| *O. europea1 - O. europea3* | 4.459 | 0.203 | n.s. |
| *O. europea1 - O. europea4* | 6.354 | 0.203 | n.s. |
| *O. europea2 - O. europea3* | 1.621 | 0.241 | n.s. |
| *O. europea2 - O. europea4* | 0.108 | 0.902 | n.s. |
| *O. europea3 - O. europea4* | 1.426 | 0.241 | n.s. |
|  |  |  |  |
| ***C. capitata*** |  |  |  |
| *F. carica1 - P. communis* | 4.856 | 0.150 | n.s. |
| *F. carica1 - F. carica2* | 6.623 | 0.150 | n.s. |
| *F. carica1 - C. reticulata* | 0.731 | 0.599 | n.s. |
| *P. communis - F. carica2* | 0.555 | 0.698 | n.s. |
| *P. communis - C. reticulata* | 4.286 | 0.150 | n.s. |
| *F. carica2 - C. reticulata* | 6.041 | 0.150 | n.s. |
|  |  |  |  |
| ***C. quilicii*** |  |  |  |
| *H. caffrum - E. japonica1* | 0.545 | 1.000 | n.s. |
| *H. caffrum - P. guajava* | 0.686 | 0.601 | n.s. |
| *H. caffrum - E. japonica2* | 1.677 | 0.452 | n.s. |
| *E. japonica1 - P. guajava* | 5.318 | 0.202 | n.s. |
| *E. japonica1 - E. japonica2* | 9.598 | 0.202 | n.s. |
| *P. guajava - E. japonica2* | 3.550 | 0.202 | n.s. |
