# Supplementary material for: Comparative Microbiomics of Tephritid Frugivorous Pests (Diptera: Tephritidae) From the Field: A Tale of High Variability Across and Within Species
Source: Front Microbiol. 2020 Aug 11;11:1890. doi: 10.3389/fmicb.2020.01890 (PMC7431611; doi:10.3389/fmicb.2020.01890)
Supplement: TABLE S9 — A posteriori pairwise comparisons (permutational t-statistics) for the significant effects detected by the PERMDISP test reported in Table 2 (presence-absence data; dataset A). ‘***’ = p < 0.001, ‘**’ = p < 0.01, ‘*’ = p < 0.05, ‘n.s.’ = p > 0.05. [file Table_9.DOCX]

Supplementary Table S9: *A posteriori* pairwise comparisons (permutational t-statistics) for the significant effects detected by the PERMDISP test reported in Table 2 (presence-absence data). ‘***’ = p < 0.001, ‘**’ = p < 0.01, ‘*’ = p < 0.05, ‘n.s.’ = p > 0.05

| **Fruit fly Species** |  |  |  |
| --- | --- | --- | --- |
| Groups | t | p-value |  |
| *B. dorsalis - Z. cucurbitae* | 0.766 | 0.644 | n.s. |
| *B. dorsalis - B. oleae* | 4.143 | 0.097 | n.s. |
| *B. dorsalis - C. capitata* | 0.185 | 0.800 | n.s. |
| *B. dorsalis - C. quilicii* | 3.014 | 0.115 | n.s. |
| *Z. cucurbitae - B. oleae* | 3.631 | 0.097 | n.s. |
| *Z. cucurbitae - C. capitata* | 0.648 | 0.644 | n.s. |
| *Z. cucurbitae - C. quilicii* | 2.507 | 0.144 | n.s. |
| *B. oleae - C. capitata* | 4.294 | 0.097 | n.s. |
| *B. oleae - C. quilicii* | 0.597 | 0.667 | n.s. |
| *C. capitata - C. quilicii* | 3.043 | 0.115 | n.s. |
|  |  |  |  |
| **Location** |  |  |  |
| Groups | t | p-value |  |
| ***B. dorsalis*** |  |  |  |
| Tanzania - South Africa | 0.809 | 0.439 | n.s. |
|  |  |  |  |
| ***Z. cucurbitae*** |  |  |  |
| Reunion - Tanzania | 2.550 | 0.004 | ** |
|  |  |  |  |
| ***B. oleae*** |  |  |  |
| Italy - Greece | 0.906 | 0.381 | n.s. |
|  |  |  |  |
| ***C. capitata*** |  |  |  |
| Italy - Greece | 0.846 | 0.439 | n.s. |
|  |  |  |  |
| ***C. quilicii*** |  |  |  |
| South Africa - Reunion | 2.446 | 0.035 | * |
|  |  |  |  |
| **Host plant** |  |  |  |
| Groups | t | p-value |  |
| ***B. dorsalis*** |  |  |  |
| *A. muricata - P. guajava* | 1.152 | 0.719 | n.s. |
| *A. muricata - M. indica* | 2.177 | 0.304 | n.s. |
| *A. muricata - E. japonica* | 1.780 | 0.304 | n.s. |
| *P. guajava - M. indica* | 0.909 | 0.719 | n.s. |
| *P. guajava - E. japonica* | 0.225 | 0.902 | n.s. |
| *M. indica - E. japonica* | 0.781 | 0.719 | n.s. |
|  |  |  |  |
| ***Z. cucurbitae*** |  |  |  |
| *C. grandis - M. charantia* | 0.060 | 1.000 | n.s. |
| *C. grandis - C. lanatus* | 1.696 | 0.601 | n.s. |
| *C. grandis - C. sativus* | 1.756 | 0.598 | n.s. |
| *M. charantia - C. lanatus* | 1.543 | 0.601 | n.s. |
| *M. charantia - C. sativus* | 1.267 | 0.601 | n.s. |
| *C. lanatus - C. sativus* | 0.811 | 0.601 | n.s. |
|  |  |  |  |
| ***B. oleae*** |  |  |  |
| *O. europea1 - O. europea2* | 0.078 | 0.801 | n.s. |
| *O. europea1 - O. europea3* | 2.232 | 0.299 | n.s. |
| *O. europea1 - O. europea4* | 2.362 | 0.299 | n.s. |
| *O. europea2 - O. europea3* | 1.426 | 0.398 | n.s. |
| *O. europea2 - O. europea4* | 1.343 | 0.450 | n.s. |
|  |  |  |  |
| ***C. capitata*** |  |  |  |
| *F. carica1 - P. communis* | 3.949 | 0.303 | n.s. |
| *F. carica1 - F. carica2* | 1.114 | 0.601 | n.s. |
| *F. carica1 - C. reticulata* | 1.862 | 0.400 | n.s. |
| *P. communis - F. carica2* | 0.555 | 0.843 | n.s. |
| *P. communis - C. reticulata* | 1.971 | 0.303 | n.s. |
| *F. carica2 - C. reticulata* | 0.286 | 0.899 | n.s. |
|  |  |  |  |
| ***C. quilicii*** |  |  |  |
| *H. caffrum - E. japonica1* | 2.784 | 0.299 | n.s. |
| *H. caffrum - P. guajava* | 0.410 | 0.700 | n.s. |
| *H. caffrum - E. japonica2* | 0.496 | 0.700 | n.s. |
| *E. japonica1 - P. guajava* | 1.748 | 0.603 | n.s. |
| *E. japonica1 - E. japonica2* | 2.359 | 0.299 | n.s. |
