# Supplementary material for: Comparative Microbiomics of Tephritid Frugivorous Pests (Diptera: Tephritidae) From the Field: A Tale of High Variability Across and Within Species
Source: Front Microbiol. 2020 Aug 11;11:1890. doi: 10.3389/fmicb.2020.01890 (PMC7431611; doi:10.3389/fmicb.2020.01890)
Supplement: TABLE S10 — A posteriori pairwise comparisons (Student-Newman-Keuls test) for the significant effects detected by the ANOVA test reported in Table 3. ‘***’ = p < 0.001, ‘**’ = p < 0.01, ‘*’ = p < 0.05, ‘n.s.’ = p > 0.05 (Dataset A). [file Table_10.DOCX]

Supplementary Table S10: *A posteriori* pairwise comparisons (Student-Newman-Keuls test) for the significant effects detected by the ANOVA test reported in Table 3. ‘***’ = p < 0.001, ‘**’ = p < 0.01, ‘*’ = p < 0.05, ‘n.s.’ = p > 0.05

| Pairwise comparisons FFSp |  |  | | |  | |  | |  |
| --- | --- | --- | --- | --- | --- | --- | --- | --- | --- |
|  | B. oleae | C. quilicii | | | B. dorsalis | | C. capitata | | Z. cucurbitae |
| Ranked means: | 0.2339 | 0.6349 | | | 0.6805 | | 0.7145 | | 0.8174 |
|  |  |  | | |  | |  | |  |
| *B. dorsalis - Z. cucurbitae* | n.s. | |  |  | |  | |  |  |
| *B. dorsalis - B. oleae* | * | |  |  | |  | |  |  |
| *B. dorsalis - C. capitata* | n.s. | |  |  | |  | |  |  |
| *B. dorsalis - C. quilicii* | n.s. | |  |  | |  | |  |  |
| *Z. cucurbitae - B. oleae* | * | |  |  | |  | |  |  |
| *Z. cucurbitae - C. capitata* | n.s. | |  |  | |  | |  |  |
| *Z. cucurbitae - C. quilicii* | n.s. | |  |  | |  | |  |  |
| *B. oleae - C. capitata* | n.s. | |  |  | |  | |  |  |
| *B. oleae - C. quilicii* | * | |  |  | |  | |  |  |
| *C. capitata - C. quilicii* | n.s. | |  |  | |  | |  |  |
|  |  |  | | |  | |  | |  |
| Standard error = 0.0942 |  |  | | |  | |  | |  |
| Df = 5 |  |  | | |  | |  | |  |
|  |  |  | | |  | |  | |  |
| Pairwise comparisons Ho within FFSp:Lo |  |  | | |  | |  | |  |
| ***B. dorsalis* - Tanzania** |  |  | | |  | |  | |  |
|  | *A. muricata* | *P. guajava* | | |  | |  | |  |
| Ranked means: | 0.7898 | 0.8807 | | |  | |  | |  |
| *A. muricata - P. guajava* | n.s. | |  |  | |  | |  |  |
|  |  |  | | |  | |  | |  |
| ***B. dorsalis* - South Africa** |  |  | | |  | |  | |  |
|  | *M. indica* | *E. japonica* | | |  | |  | |  |
| Ranked means: | 0.4067 | 0.6448 | | |  | |  | |  |
| *M. indica - E. japonica* | n.s. | |  |  | |  | |  |  |
|  |  |  | | |  | |  | |  |
| ***Z. cucurbitae* - Reunion** |  |  | | |  | |  | |  |
|  | *C. grandis* | *M. charantia* | | |  | |  | |  |
| Ranked means: | 0.7635 | 0.8079 | | |  | |  | |  |
| *C. grandis - M. charantia* | n.s. | |  |  | |  | |  |  |
|  |  |  | | |  | |  | |  |
| ***Z. cucurbitae* - Tanzania** |  |  | | |  | |  | |  |
|  | *C. sativus* | *C. lanatus* | | |  | |  | |  |
| Ranked means: | 0.7959 | 0.9023 | | |  | |  | |  |
| *C. lanatus - C. sativus* | n.s. | |  |  | |  | |  |  |
|  |  |  | | |  | |  | |  |
| ***B. oleae* - Italy** |  |  | | |  | |  | |  |
|  | *O. europea2* | *O. europea1* | | |  | |  | |  |
| Ranked means: | 0.1325 | 0.5845 | | |  | |  | |  |
| *O. europea1 - O. europea2* | ** | |  |  | |  | |  |  |
|  |  |  | | |  | |  | |  |
| ***B. oleae* - Greece** |  |  | | |  | |  | |  |
|  | *O. europea4* | *O. europea3* | | |  | |  | |  |
| Ranked means: | 0.0822 | 0.1365 | | |  | |  | |  |
| *O. europea3 - O. europea4* | n.s. | |  |  | |  | |  |  |
|  |  |  | | |  | |  | |  |
| ***C. capitata* - Italy** |  |  | | |  | |  | |  |
|  | *P. communis* | *F. carica1* | | |  | |  | |  |
| Ranked means: | 0.714 | 0.7861 | | |  | |  | |  |
| *F. carica1 - P. communis* | n.s. | |  |  | |  | |  |  |
|  |  |  | | |  | |  | |  |
| ***C. capitata* - Greece** |  |  | | |  | |  | |  |
|  | *C. reticulata* | *F. carica2* | | |  | |  | |  |
| Ranked means: | 0.6376 | 0.7204 | | |  | |  | |  |
| *F. carica2 - C. reticulata* | n.s. | |  |  | |  | |  |  |
|  |  |  | | |  | |  | |  |
| ***C. quilicii* - South Africa** |  |  | | |  | |  | |  |
|  | *H. caffrum* | E. japonica1 | | |  | |  | |  |
| Ranked means: | 0.2641 | 0.9022 | | |  | |  | |  |
| *H. caffrum - E. japonica1* | *** | |  |  | |  | |  |  |
|  |  |  | | |  | |  | |  |
| ***C. quilicii* - Reunion** |  |  | | |  | |  | |  |
|  | *E. japonica2* | *P. guajava* | | |  | |  | |  |
| Ranked means: | 0.6771 | 0.6962 | | |  | |  | |  |
| *P. guajava - E. japonica2* | n.s. | |  |  | |  | |  |  |
|  |  |  | | |  | |  | |  |
| Standard error = 0.093 |  |  | | |  | |  | |  |
| Df = 40 |  |  | | |  | |  | |  |
