# Supplementary material for: Comparative Microbiomics of Tephritid Frugivorous Pests (Diptera: Tephritidae) From the Field: A Tale of High Variability Across and Within Species
Source: Front Microbiol. 2020 Aug 11;11:1890. doi: 10.3389/fmicb.2020.01890 (PMC7431611; doi:10.3389/fmicb.2020.01890)
Supplement: TABLE S11 — Most abundant bacterial genera observed in each fruit fly species (% as estimated from number of reads). [file Table_11.DOCX]

Supplementary Table S11: Most abundant bacterial genera observed in each fruit fly species (% as estimated from number of reads).

| *B. dorsalis* | *Z. cucurbitae* | *B. oleae* | *B. zonata* | *C. capitata* |
| --- | --- | --- | --- | --- |
| *Enterobacter*  (37.96%) | *Ochrobactrum* (20.35%) | *Erwinia* (93.28%) | *Enterobacter* (31.15%) | *Morganella* (32.5%) |
| *Klebsiella*  (27.36%) | *Citrobacter* (13.84%) | *Pantoea* (4.97%) | *Klebsiella* (26.3%) | *Pantoea* (18.61%) |
| *Leuconostoc* (10.52%) | *Klebsiella* (12.83%) | *Pseudomonas* (0.56%) | *Lactococcus* (22.63%) | *Klebsiella* (11.71%) |
| *Providencia* (6.87%) | *Lactococcus* (10.97%) | *Rahnella* (0.33%) | *Leuconostoc* (2.82%) | Enterobacteriaceae sp. (7.97%) |
| Enterobacteriaceae sp. (5.97%) | *Raoultella* (5.46%) | *Lelliottia* (0.23%) | *Morganella* (2.41%) | *Fructobacillus* (6.87%) |
| *Kluyvera* (1.88%) | *Enterobacter* (5.33%) | Enterobacteriaceae sp. (0.08%) | Enterobacteriaceae sp. (2.27%) | *Gluconobacter* (5.39%) |
| *Morganella* (1.42%) | Enterobacteriaceae sp. (4.48%) | *Bacillus* (0.08%) | *Serratia* (1.56%) | *Enterobacter*  (3.1%) |
| *Lactobacillus* (1.24%) | *Chishuiella* (2.89%) | *Citrobacter* (0.07%) | *Dysgonomonas* (1.49%) | *Acetobacter* (2.55%) |
| *Lactococcus* (0.79%) | *Providencia* (2.12%) | *Sphingomonas* (0.07%) | *Weissella* (1.47%) | *Zymobacter* (2.01%) |
| *Acetobacter* (0.76%) | *Acinetobacter* (1.97%) | *Escherichia*/*Shigella* (0.04%) | *Providencia* (1.2%) | *Providencia* (1.81%) |
|  |  |  |  |  |
|  |  |  |  |  |
| *C. quilicii* | ***C. rosa*** | ***C. cosyra*** | ***C. flexuosa*** | ***C. podocarpi*** |
| *Gluconobacter* (30%) | *Acetobacter* (55.31%) | *Klebsiella* (24.96%) | *Providencia* (31.72%) | *Klebsiella* (52.83%) |
| *Commensalibacter* (13.87%) | *Gluconobacter* (29.81%) | *Gluconobacter* (15.95%) | *Commensalibacter* (30.61%) | *Rahnella* (17.7%) |
| *Acetobacter* (10.04%) | *Enterobacteriaceae* sp. (10.16%) | *Enterobacter* (14.48%) | *Morganella* (29.31%) | *Staphylococcus* (5.8%) |
| *Tatumella* (8.72%) | *Candidatus*_*Schmid*-*hempelia* (2.16%) | *Pantoea* (11.26%) | *Enterobacter* (2.33%) | Enterobacteriaceae sp. (4.09%) |
| *Providencia* (7.94%) | *Asaia* (1.18%) | *Providencia* (9.72%) | *Leuconostoc* (1.54%) | *Curvibacter* (2.52%) |
| *Serratia* (5.65%) | *Tatumella* (0.47%) | *Weissella* (8.61%) | *Rhodococcus* (0.74%) | *Enhydrobacter* (2.08%) |
| *Morganella* (4.78%) | *Bacillus* (0.29%) | *Acetobacter* (5.95%) | *Weissella* (0.42%) | *Corynebacterium* (1.8%) |
| *Komagataeibacter* (3.74%) | Escherichia/*Shigella* (0.23%) | Enterobacteriaceae sp. (2.15%) | *Bacillus* (0.36%) | *Streptococcus* (1.64%) |
| Enterobacteriaceae sp. (2.1%) | *Serratia* (0.06%) | *Leuconostoc* (1.29%) | *Pseudarthrobacter* (0.27%) | *Acinetobacter* (1.52%) |
| *Lactobacillus* (1.58%) | *Pluralibacter* (0.06%) | *Tatumella* (1.18%) | *Escherichia*/*Shigella* (0.18%) | *Enterobacter* (1.24%) |
